# Comparison of the efficacy and safety of tocilizumab for colchicine-resistant or colchicine-intolerant familial Mediterranean fever: study protocol for an investigator-initiated, multicenter, randomized, double-blind, placebo-controlled trial

**DOI:** 10.1186/s13063-018-3105-6

**Published:** 2018-12-29

**Authors:** Tomohiro Koga, Shuntaro Sato, Junya Miyamoto, Naoko Hagimori, Yurika Kawazoe, Kumiko Arinaga, Chizu Fukushima, Hiroshi Yamamoto, Atsushi Kawakami

**Affiliations:** 1 Department of Immunology and Rheumatology, Division of Advanced Preventive Medical Sciences, Nagasaki University Graduate School of Biomedical Sciences, 1-7-1 Sakamoto, Nagasaki, 852-8501 Japan; 2 Center for Bioinformatics and Molecular Medicine, Nagasaki University Graduate School of Biomedical Sciences, 1-7-1 Sakamoto, Nagasaki, 852-8523 Japan; 3 Nagasaki University Hospital, Clinical Research Center, 1-7-1 Sakamoto, Nagasaki, 852-8501 Japan; 4 Center for Bioinformatics and Molecular Medicine, Nagasaki University Graduate School of Biomedical Sciences, 1-12-4 Sakamoto, Nagasaki, 852-8523 Japan

**Keywords:** IL-6, Placebo, Tocilizumab, FMF, Colchicine-resistant

## Abstract

**Background:**

Familial Mediterranean fever (FMF) is an inherited disorder caused by a number of mutations of the Mediterranean fever (*MEFV*) gene, coding a protein named pyrin that acts as a major regulatory component of the inflammasome. The first-line drug for FMF treatment is colchicine, but 10% of patients with FMF do not respond well to colchicine. Although the efficacy of tocilizumab (TCZ), which is a recombinant, humanized, antihuman interleukin 6 (IL-6) receptor monoclonal antibody, has been reported to prevent FMF attacks, the effects of TCZ on individuals with colchicine-resistant or colchicine-intolerant FMF have not been evaluated in a randomized clinical trial.

**Methods/design:**

In this phase III, investigator-initiated, multicenter, double-blind, randomized, parallel-group trial, the efficacy and safety of TCZ will be compared with placebo in patients with colchicine-resistant or colchicine-intolerant FMF. The study will be conducted in nine centers in Japan. Participants (*n* = 24) will be randomly assigned to receive 162 mg of TCZ (*n* = 12) or placebo (*n* = 12) administered subcutaneously once weekly for 24 weeks. Rescue treatment will be allowed if rescue criteria are met. A primary endpoint is the number of fever attacks until 24 weeks. Secondary endpoints include the number of occurrences of accompanying symptoms during attacks; the time until a fever attack occurs; the duration of fever attacks; serum C-reactive protein and serum amyloid A; 36-item Short Form Health Survey; general evaluation by a physician (100-mm visual analogue scale); body temperature; the percentage of subjects who achieve FMF 50 at 12 weeks and 24 weeks; and pharmacodynamic assessment, including the measurement of serum TCZ level and soluble IL-6 receptor.

**Discussion:**

The study is expected to produce evidence regarding the efficacy of a potential new therapeutic agent, TCZ, in improving the clinical course and outcome for patients with colchicine-resistant or colchicine-intolerant FMF.

**Trial registration:**

University Hospital Medical Information Network Clinical Trials Registry, UMIN000028010. Registered on 7 July 2017.

**Electronic supplementary material:**

The online version of this article (10.1186/s13063-018-3105-6) contains supplementary material, which is available to authorized users.

## Background

Familial Mediterranean fever (FMF) is an inherited autoinflammatory disorder characterized by recurrent attacks of fever with arthritis, abdominal pain, skin rash, and/or serositis [1, 2]. FMF is an autosomal recessive disorder, and it is most common in the Mediterranean region, especially among Turks, Armenians, non-Ashkenazi Jews, and Arabs; however, patients with FMF were also recently reported in Japan, where the population has genetic characteristics that are different from those observed in the endemic areas [3].

FMF is caused by a number of mutations of the Mediterranean fever (*MEFV*) gene, coding a protein named pyrin that acts as a major regulatory component of the inflammasome [4]. Accordingly, the pathological condition of FMF is thought to be mainly an abnormal activation of the inflammasome caused by mutations of pyrin, but refractory cases occur despite the absence of genetic mutations in the *MEFV* gene.

The most common symptom of FMF is periodic fever. Typical cases have a short fever duration of 1–3 days, and fevers are spontaneously alleviated. There are individual differences in the frequency of fever attacks, and there are various stressors that may precede a fever attack, such as the invasiveness of surgery and menstruation. In addition, although they are less frequent, epicarditis, aseptic meningitis, and erysipelas-like skin lesion are sometimes observed. The most serious complication is amyloid A amyloidosis.

The goals of treatment for FMF are to prevent acute attacks and minimize subclinical inflammation between attacks, which leads to the progression of amyloidosis. Colchicine is effective as a prophylactic treatment for FMF attacks and is recommended as the first-line drug in adults and children. However, 10% of patients with FMF do not respond well to colchicine or are not able to continue this drug because of adverse effects. Accordingly, these colchicine-resistant or colchicine-intolerant patients with FMF need other treatments. As another therapy, an interleukin 1 (IL-1) inhibitor can be considered as an alternative agent. Canakinumab, an IL-1β inhibitor, was approved for FMF in Japan in December 2016. Although studies and clinical trials have revealed the efficacy of canakinumab [5], there is little evidence regarding the efficacy and safety of canakinumab in Japanese patients with FMF.

Patients with FMF have been reported to show increased serum levels of inflammatory cytokines such as IL-1β, IL-6, IL-17, and IL-18 [6–8]. With the use of a multisuspension cytokine array, we recently revealed the specific cytokine network in patients with FMF [9]. That study showed that IL-6 had the best performance for distinguishing FMF in attack from healthy control subjects or FMF in remission. If the participants meet the inclusion criteria at this point, they will be randomized. In line with these observations, other case reports have shown the efficacy of an IL-6 inhibitor in clinical practice for colchicine-resistant FMF or secondary amyloidosis in patients with FMF [10–13]. Taken together, these findings enabled us to design the current phase III study to confirm the beneficial effects of tocilizumab (TCZ) in patients with FMF. In this article, we describe the final protocol (version 3.1; 19 April 2018) for this study. The results of this study are expected to provide evidence regarding the usefulness of TCZ for the treatment of colchicine-resistant or colchicine-intolerant patients with FMF.

## Methods/design

### Study design

**Fig. 1:**
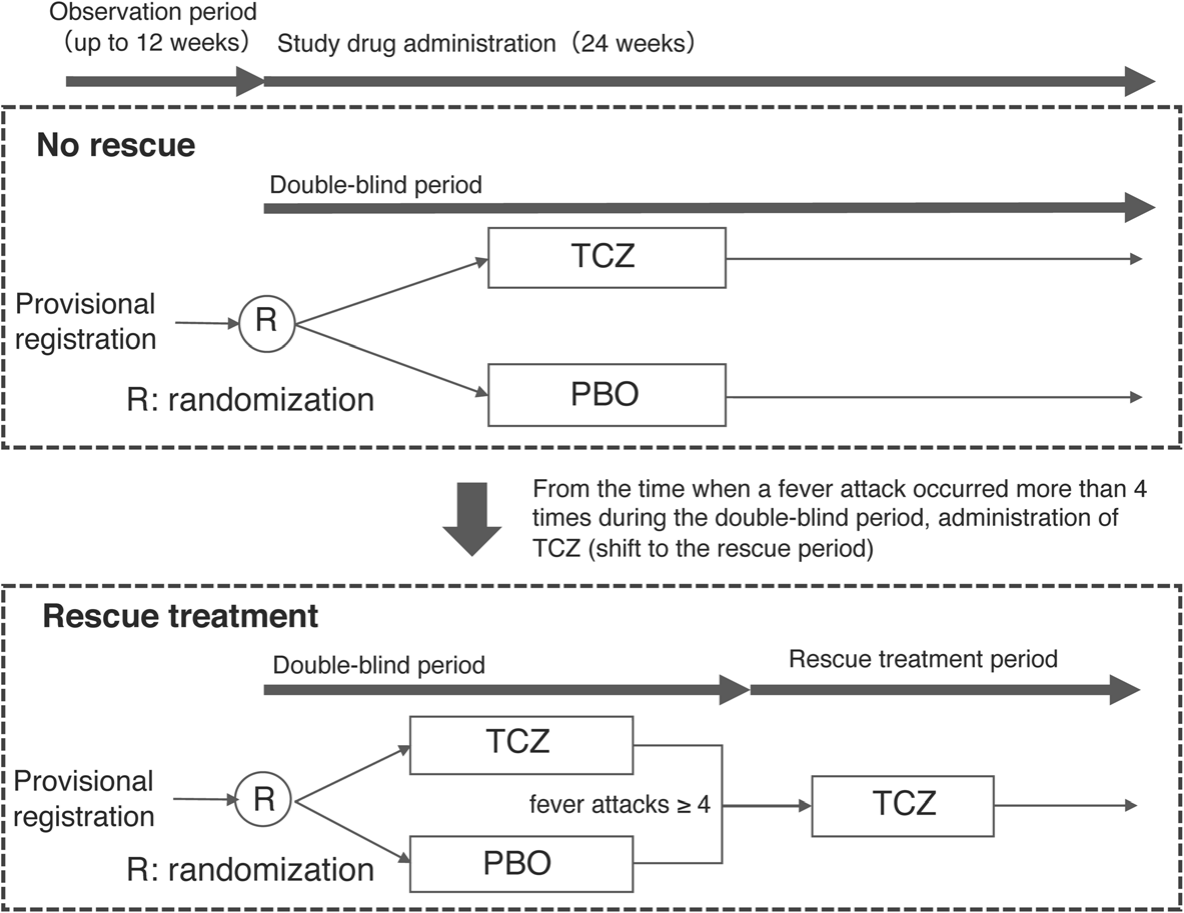
Study design. *PBO* Placebo, *TCZ* Tocilizumab

The present study design is in accordance with the Standard Protocol Items: Recommendations for Interventional Trials (SPIRIT) and Consolidated Standards of Reporting Trials 2010 guidelines [14, 15] (*see* Additional file 1). This is an investigator-initiated, multicenter, phase III, double-blind, randomized, parallel-group comparison study of the efficacy and safety of TCZ compared with placebo in patients with colchicine-resistant or colchicine-intolerant FMF. The study design is summarized in Fig. 1.

The study will be conducted at nine centers in Japan. The study is registered with the University Hospital Medical Information Network Clinical Trials Registry (www.umin.ac.jp/ctr/) under registration number UMIN000028010. We will conduct the study in accordance with the principles of the Declaration of Helsinki [16] and Japanese Ministerial Ordinance on Good Clinical Practice. The local ethics committee at each center will approve the study (approvals are already in place; *see* Additional file 2).

### Participant recruitment

Participants will be recruited at Nagasaki University Hospital, Kyushu University Hospital, Kyoto University Hospital, Yokohama City University Hospital, Chiba University Hospital, Kanazawa University Hospital, Shinshu University Hospital, Fukushima Medical University, and Hokkaido University Hospital. All eligible patients will be selected and approached on the basis of information derived from the electronic health records of these nine hospitals according to the inclusion and exclusion criteria. In addition, we mailed a letter describing the outline of this clinical trial to the institutions and stating that we had been requested to analyze the *MEFV* gene. Participants will be provided with an explanation about the study by their treating pediatricians/rheumatologists and the clinical research coordinator (CRC), and they will be asked to voluntarily sign an informed consent before participation.

### Inclusion criteria

The criteria for provisional registration are as follows:

1. Patients diagnosed with FMF (clinically typical) according to the following diagnostic criteria (“regarding diagnostic criteria and severity classification related to designated intractable diseases” by the Ministry of Health, Labor and Welfare in Japan)
2. Patients with colchicine-ineffective or colchicine-inadequate responses
3. Patients aged 12 to 75 years old (regardless of gender)
4. Patients who received a thorough explanation of the contents of explanatory documents and other matters concerning clinical trials, and who understand the contents thereof, and who provide written consent based on their free will to participate in this trial

After the provisional registration, patients who have had a fever attack due to FMF (a fever that lasted > 6 h and had a fever > 38.0 °C) during up to 12 weeks of the observation period are randomly assigned to the TCZ group or the placebo group.

### Exclusion criteria

The exclusion criteria are as follows: women who are breastfeeding, pregnant, or may become pregnant; obvious infection within 4 weeks before the study and considered inappropriate by an investigator or clinical trial physician; a history of hypersensitivity to the components of TCZ; recent treatment with a biologic: a history of TCZ treatment; routine use of corticosteroids (excluding topical therapy such as external preparations) for diseases other than FMF; and judged as inappropriate by the clinical investigator or clinical trial physician for any other reason.

After the provisional registration, we will examine each patient’s laboratory test results obtained within 1 week prior to the initial administration and will exclude patients who meet the following criteria: leukocyte count < 4000/μl, neutrophil count < 1000/μl, lymphocyte count < 500/μl, number of platelets < 100,000/μl, active tuberculosis, a history of intestinal perforation, interstitial pneumonia and judged inappropriate by the investigator or clinical trial physician, a malignant tumor within 5 years before the study, active type B or C hepatitis, complicated serious diseases, inoculated with a live vaccine within 6 weeks before the study, or use of other investigational drugs within 6 months before the initial investigational drug.

### Rescue treatment

Rescue treatment will be allowed during the trial if the patient meets all of the following five criteria:

1. Patients who received one or more investigational drugs in a double-blind period
2. Patients who have experienced more than four fever attacks since the start of investigational drug administration
3. Patients whose fever attacks of criterion 2 have disappeared
4. Patients judged by investigators or clinical trial physicians to be appropriate from a safety point of view
5. Patients who provide consent via another person (i.e., subjects < 20 years of age)

### Randomization and blinding

After the acquisition of written informed consent and the completion of baseline measurements, the enrolled participants are registered and allocated by facsimile using the system of the participant’s registration center. Participants will be randomly allocated at a ratio of 1:1 (TCZ vs. placebo). We have not set the assignment factor for this trial. Randomization will be performed using a block randomization method with SAS version 9.4 software (SAS Institute, Cary, NC, USA). The allocation is kept in opaque, sequentially numbered envelopes; envelopes are sequentially transferred to each hospital and assigned by the pharmacist to each next patient. Patients, physicians, nurses, researchers, and data analyst/statistician will be blinded to treatment allocation until study completion. In order to maintain blindness, measurements of C-reactive protein (CRP), serum amyloid A (SAA), and sedimentation, which directly reflect the efficacy of IL-6, are prohibited during double-blind periods, except in the case of an emergency such as development of an infectious disease.

### Study protocol

**Fig. 2:**
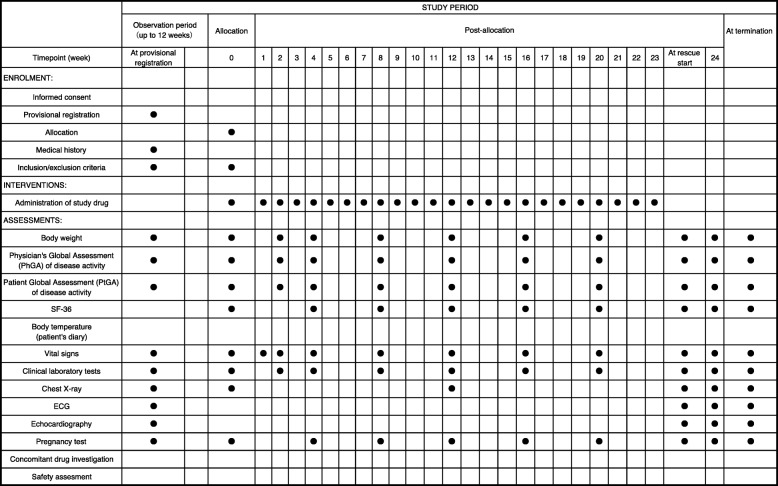
Treatment schedule and outcome measures. *ECG* Electrocardiogram

**Fig. 3:**
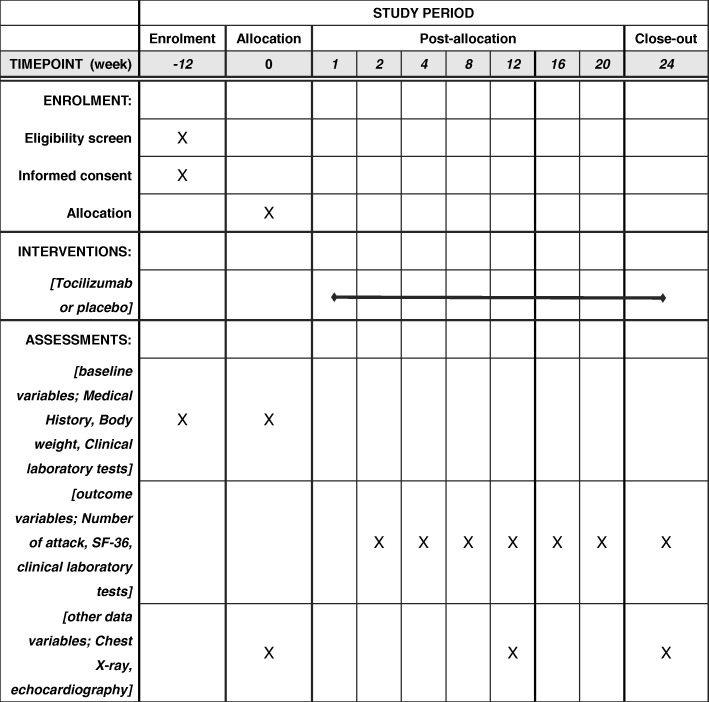
The schedule of enrollment, interventions, and assessments

A clinical trial physician will explain the study protocol to each colchicine-resistant or colchicine-intolerant patient with FMF. If the patient’s consent is obtained, a clinical trial physician will perform the observation/examination at the time of provisional registration, based on the description in Figs. 2 and 3. According to the inclusion criteria and exclusion criteria, the CRC will fax a provisional registration form to the registration center.

A patient diary will be offered to each participant on the visit date for provisional registration. The participants will be asked to record items such as daily body temperature and accompanying symptoms, including headache, arthritis, chest pain, back pain, and abdominal pain, in the diary from the day of arrival at provisional registration to the end of the trial. The participants are told that if their first fever attack occurs after the visit at the time of provisional registration, they should consult the appropriate medical institution within 1 week after the disappearance of the fever attack for a further evaluation of the registration (randomization).

After confirming that a patient meets the inclusion criteria and does not meet any of the exclusion criteria, the investigators send the patient’s registration card to the registration center and administer the first investigational drug. If the participant does not have fever attacks during the 12-week period from the time of the provisional registration visit, he or she is not registered in the trial. However, if provisional registration is possible within the study period (by December 31, 2018), the provisional registration is possible again.

Patients were randomly assigned 1:1 to receive weekly TCZ 162 mg or placebo subcutaneously. After the visit date (i.e., the initial investigation day of the investigational drug), the investigators will continue to administer the investigational drug and conduct necessary examinations and surveys in accordance with the schedule shown in Fig. 2. If fever attacks occur more than four times during the double-blind period, a clinical trial physician will confirm whether the patient’s case meets the criteria at the time of rescue treatment, and the physician will introduce TCZ subcutaneous injection after the disappearance of the most recent fever attack. The observation/examination will be terminated 24 weeks after the initial investigational drug administration period in the double-blind period. During the double-blind period, patients and investigators will be blinded to the CRP and SAA results because TCZ can directly affect the levels of CRP and SAA.

### Adverse events

All serious adverse events (SAEs) that occur between the signing of informed consent and the end of week 24 in the double-blind period will be recorded. Events occurring after the end of week 24 in the double-blind period will be monitored during an open-label continuation trial. An SAE is defined as any untoward medical occurrence that occurs at any dose, results in death, is life-threatening, requires inpatient hospitalization or prolongation of existing hospitalization, results in persistent or significant disability or incapacity, or causes a congenital anomaly or birth defect.

The study design incorporates an independent data monitoring committee that will review the ongoing safety data in an unblinded manner in accordance with the standard operating procedures of the Center for Clinical Trials, Japan Medical Association (www.jmacct.med.or.jp/).

### Outcome measurements

The primary endpoint is the number of fever attacks until 24 weeks. The definition of “fever attack” in this clinical trial is a fever > 38.0 °C lasting ≥ 6 h. When a fever attack occurs, it is necessary to confirm that the cause of the fever and accompanying symptoms described in the patient’s diary is not a disease other than FMF, such as an infectious disease. The secondary endpoints are efficacy, safety, and exploratory categories.

### Efficacy

We will evaluate the efficacy of the investigational drug on basis of the following parameters: the number of occurrences of accompanying symptoms during attacks, the time until a fever attack occurs, the duration of fever attacks, the serum CRP and SAA, the patient’s score on the 36-item Short Form Health Survey, results of a general evaluation by a physician (100-mm visual analogue scale), body temperature, and the percentage of achievement of FMF 50 score [17] at 12 weeks and 24 weeks.

### Safety

The safety evaluation index of this clinical trial is as follows: adverse events (adverse event incidence rate, SAE incidence rate, side effect incidence rate); clinical examination (hematological examination, blood biochemical examination, urinalysis); all medically important indicators (e.g., physical findings, vital signs, electrocardiograms, echocardiography); a pharmacodynamic assessment, including measurement of the serum TCZ level and soluble IL-6 receptor.

### Exploratory

For the further evaluation including a pharmacogenomic study to predict the efficacy of this investigational drug, we will collect blood samples and maintain them under the consent of the patient separately from the consent of this trial. In principle, blood is collected once at the first fever attack of the observation period or on the first administration day.

### Data collection and management

Appropriate and authorized persons (investigators, clinical trial physicians, or clinical trial collaborators) prepare case report forms (CRFs). All data recorded in the CRF must be consistent with the original material unless the data recorded directly in the CRF are used as the source material. According to the schedule shown in Fig. 2, the investigator will collect data at each visit during the study.

The investigators will be given access to an online, web-based, electronic data capture system. Only the investigator will be able to enter and correct data in the electronic CRF (e-CRF). All study findings and documents will be regarded as confidential. Patients will be identified on the e-CRF by their patient number and/or birth date, not by name. Documents that identify the patient must be maintained in confidence by the investigator so that the anonymity of participating patients is ensured.

During the study, a sponsor-investigator will make regular site visits to review protocol compliance, conduct source data verification, assess drug accountability and management, assess laboratory procedures, and ensure that the study is being conducted according to pertinent regulatory and protocol requirements.

The study blinding should only be broken in a medical emergency (where knowledge of the study drug received would affect the treatment of the emergency) or as a regulatory requirement (e.g., for SAEs or death).

### Sample size considerations and statistical analysis

We estimated that a sample size of 24 patients (12 patients in each group) would provide ≥ 80% power for the comparison of the primary endpoint (i.e., the number of fever attacks for 24 weeks) between the TCZ and placebo groups with assumed average numbers of fever attacks of 6 and 1.5, with a two-sided alpha level of 0.05, based on the negative binomial distribution. Dropout rates during the period of investigational drug administration of the TCZ group and placebo group are assumed to be 15% and 5%, respectively. Negative binomial dispersion parameters of the TCZ group and placebo group are assumed to be 4 and 2.6, respectively.

The estimation of the sample size is based on the simulation with 10,000 repetitions using SAS version 9.4 software (SAS Institute, Cary, NC, USA). Table 1 shows the normal power based on the average number of fever attacks in the placebo group and TCZ group in the 24 cases. We will perform a negative binomial regression analysis with the fever attack number as the outcome variable, the drug (placebo or TCZ) as the explanatory variable, and the double-blind period as the offset term.

**Table 1:** Statistical power based on average number of febrile attacks

		TCZ group^a^		
		1	1.5	2
Placebo group^a^	5.5	91.5	80	65.8
	6	94.5	86.1	73.3
	6.5	96.2	90	79.8

*TCZ* Tocilizumab

^a^The average number of febrile attacks during 24 weeks in each group is shown

The full analysis set (FAS) will consist of all randomized and treated patients for whom one or more efficacy endpoints can be evaluated. The per-protocol set (PPS) will consist of patients in the FAS, excluding those with major protocol violations. We will perform statistical analyses for the primary endpoint using the FAS and secondary endpoints using both the FAS and PPS.

The safety set will consist of all patients who receive at least one dose of the study drug. The safety and tolerability analyses will be based on this analysis set. We will replace adverse events with the corresponding Medical Dictionary for Regulatory Activities code and tabulate the number of expression cases and for each event defined by the SOC and preferred term. Among them, for cases that cannot be denied causally, the number of patients and the number of events are counted separately as side effects.

Importation of data related to this clinical trial is performed using SAS version 9.2 or higher software. The dataset used for the statistical analyses will be created using the SAS program. Statistical tests will be two-sided, and *p* values < 0.05 will be accepted as significant for the primary endpoint.

## Discussion

The purpose of this clinical trial is to examine the safety and efficacy of TCZ 162 mg/week in patients with colchicine-resistant or colchicine-intolerant FMF. In order to adequately evaluate the efficacy of TCZ, a placebo was used as a double-blind parallel group comparison test. We set the period of administration of the investigational drug to 24 weeks for the following reasons. The Japan Intractable Diseases Information Center defines cases in which fever attacks are observed more than four times annually as “frequent fever attack” cases; the intervals between fever attacks vary and are different in individual patients. In this trial, fever attacks are expected to occur frequently, especially in the placebo group. Therefore, we allow a rescue shift to TCZ when exacerbation attacks occur more than four times. The rescue period in clinical trials with biologic agents for rheumatoid arthritis (RA) has often been set as within 3 to 4 months [18]. Because patients with severe FMF present frequent fever attacks (e.g., once per month), it is appropriate to start the rescue period after four or more fever attacks in this trial.

In this trial, we decided to administer the investigational drug once per week (qw). There have been no reports determining the optimal dosage of TCZ for FMF. This drug has been approved in Japan for Takayasu arteritis (TA) and giant cell arteritis (GCA) by subcutaneous injection of TCZ (SC-TCZ) qw, and it has also been approved in Japan to shorten the dosing interval to qw among patients with RA who have an inadequate response to TCZ-SC every other week (q2w). It has been demonstrated that a patient’s serum IL-6 concentration before TCZ administration predicts the efficacy of TCZ in patients with RA, suggesting that the serum IL-6 concentration as a ligand of TCZ may be related to the required amount [19]. Our recent data showed that there is no significant difference in the serum IL-6 concentration between patients with active RA and patients with FMF during attack [20]. We selected SC-TCZ qw to eliminate the possibility of insufficient exposure to the drug and to obtain better effects than q2w. We have already observed safe SC-TCZ qw administration for patients with RA, TA, or GCA.

Regarding the evaluation method, we will evaluate the frequency of fever attacks and accompanying symptoms that are the most prominent and clinically important. However, taking into consideration the ethical requirements, after a fourth fever attack appears, the patient will be shifted to rescue treatment (the administration of open-label TCZ). The purpose of the above-described sample size calculation is to determine the optimal number of participants to be included in the trial; therefore, we set the number of patients needed as 24, because we speculate that the average number of fever attacks over the 24 weeks will be 5 in the placebo group and 1.5 in the TCZ group. According to a consensus with an autoinflammatory disease specialist, we suspect that an exacerbation attack will occur at a frequency of once per month among patients with active FMF, and the expected number of fever attacks over 24 weeks is calculated as approximately six times for placebo. TCZ administration has been described only in case reports, but those reports noted that in most cases, the fever attacks disappeared with TCZ treatment [5, 13]. Accordingly, it is reasonable to set the average number of fever attacks at 1.5 over 24 weeks.

This trial will evaluate the efficacy and safety of TCZ in colchicine-resistant or colchicine-intolerant patients with FMF. It will also add to the understanding of the potential efficacy of IL-6 inhibitors on secondary amyloidosis, which may dramatically improve the prognoses of individuals with FMF. The findings will provide new therapeutic options for severe FMF.

## Trial status

The trial started on 1 March 2018 and is currently recruiting.

## Additional files

Additional file 1: SPIRIT 2013 checklist: recommended items to address in a clinical trial protocol and related documents. (DOC 121 kb)

Additional file 2: List of Ethical Committee approvals. (DOCX 65 kb)

## Abbreviations

CRC: Clinical research coordinator
CRF: Case report form
CRP: C-reactive protein
FAS: Full analysis set
FMF: Familial Mediterranean fever
GCA: Giant cell arteritis
IL: Interleukin
*MEFV*: Mediterranean fever gene
PPS: Per-protocol set
q2w: Every other week
qw: Once per week
RA: Rheumatoid arthritis
SAA: Serum amyloid A
SAEs: Serious adverse events
SPIRIT: Standard Protocol Items: Recommendations for Interventional Trials
TA: Takayasu arteritis
TCZ: Tocilizumab
