# Supplementary material for: Comparison of the efficacy and safety of tocilizumab for colchicine-resistant or colchicine-intolerant familial Mediterranean fever: study protocol for an investigator-initiated, multicenter, randomized, double-blind, placebo-controlled trial
Source: Trials. 2018 Dec 29;19:715. doi: 10.1186/s13063-018-3105-6 (PMC6311086; doi:10.1186/s13063-018-3105-6)
Supplement: Supplementary file 2 — List of Ethical Committee approvals. (DOCX 65 kb) [file 13063_2018_3105_MOESM2_ESM.docx]

Additional file 2

| **country** | **Site Name** | **Date Ethics Committee Approval Received** | **Date clinical trial start** |
| --- | --- | --- | --- |
| Japan | Nagasaki University Hospital | 27-Sep-17 | 28-Sep-17 |
| Japan | Kyushu University Hospital | 28-Jun-18 | 6-Jul-18 |
| Japan | Kyoto University Hospital | 28-Feb-18 | 1-Mar-18 |
| Japan | Yokohama City University Hospital | 20-Feb-18 | 21-Feb-18 |
| Japan | Chiba University Hospital | 21-Feb-18 | 22-Feb-18 |
| Japan | Kanazawa University Hospital | 14-Mar-18 | 15-Mar-18 |
| Japan | Fukushima Medical University Hospital | 28-Feb-18 | 14-Mar-18 |
| Japan | Shinshu University Hospital | 27-Mar-18 | 28-Mar-18 |
| Japan | Hokkaido University Hospital | 19-Jun-18 | 22-Jun-18 |
